# Investigation of Mg–*x*Li–Zn alloys for potential application of biodegradable bone implant materials

**DOI:** 10.1007/s10856-021-06516-8

**Published:** 2021-04-06

**Authors:** Jingan Li, Panyu Zhou, Liguo Wang, Yachen Hou, Xueqi Zhang, Shijie Zhu, Shaokang Guan

**Affiliations:** School of Materials Science and Engineering & Henan Key Laboratory of Advanced Magnesium Alloy & Key Laboratory of Materials Processing and Mold Technology (Ministry of Education), Zhengzhou University, Zhengzhou, 450001 China

## Abstract

Implant therapy after osteosarcoma surgery is a major clinical challenge currently, especially the requirements for mechanical properties, degradability of the implants, and their inhibition of residual tumor cells. Biodegradable magnesium (Mg) alloy as medical bone implant material has full advantages and huge potential development space. Wherein, Mg–lithium (Li) based alloy, as an ultra-light alloy, has good properties for implants under certain conditions, and both Mg and Li have inhibitory effects on tumor cells. Therefore, Mg–Li alloy is expected to be applied in bone implant materials for mechanical supporting and inhibiting tumor cells simultaneously. In this contribution, the Mg–*x*Li–Zinc (Zn) series alloys (*x* = 3 wt%, 6 wt%, 9 wt%) were prepared to study the influence of different elements and contents on the structure and properties of the alloy, and the biosafety of the alloy was also evaluated. Our data showed that the yield strength, tensile strength, and elongation of as-cast Mg–*x*Li–Zn alloy were higher than those of as-cast Mg–Zn alloy; Mg–*x*Li–Zn alloy can kill osteosarcoma cells (MG-63) in a concentration-dependent manner, wherein Mg–3Li–Zn alloy (x = 3 wt%) and Mg–6Li–Zn alloy (*x* = 6 wt%) promoted the proliferation of osteoblasts (MC3T3) at a certain concentration of Li. In summary, our study demonstrated that the Mg–6Li–Zn alloy could be potentially applied as a material of orthopedic implant for its excellent multi-functions.

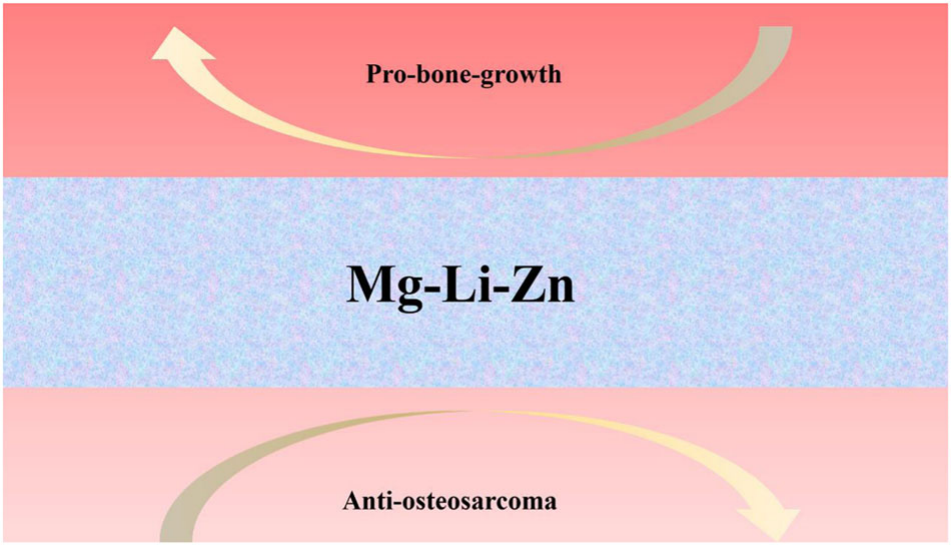

## Introduction

In recent years, the situation of fracture and bone defect caused by aging, trauma, tumor, and other factors is increasing, and the injured and diseased bone is unable to heal itself, especially in the case of tumor resection or a large number of bone defects, biodegradable materials are needed to provide temporary support during the healing process and disappear gradually by degradation after tissue recovered [1–3]. Therefore, the clinical requirements for the quality of bone-implant materials are increasingly strict: at the early stage of implantation, it can support and shape the bone to make the damaged bone tissue heal; at the later stage of implantation, it can gradually degrade in the human body; after the bone healing, it can completely degrade to avoid the pain and economic burden caused by the second operation [4]. Medical metals have been widely studied and recognized as bone implant materials because of their high strength, good toughness, excellent processing performance, and other advantages [5, 6]. Traditional medical metals for bone implants mainly include stainless steel, titanium alloy, Co–Cr alloy, etc. [7–9]. However, recent researches find that there are some disadvantages in these implants: The toxic ions, Ni and Cr ions released from stainless steel, as well as Al released from titanium alloy, which will cause irreversible damage if absorbed excessively by the human body [10–12]; The elastic modulus of these bone implants is too much higher than that of natural bone, which can not avoid stress shielding effect [13, 14]; These implants can not degrade completely by themselves, and they need to be taken out again after bone healing [15]. Therefore, it has become the focus of orthopedic clinic to find a suitable medical metal which releases no toxic ions, produce no stress shielding, and degrade at the same time.

Recently, magnesium (Mg) alloys have attracted rich attention and have been widely studied for some available properties, such as good mechanical property and biodegradable property which can be further improved [16–18], so they are considered as suitable choices of bone-implant materials [19, 20]. Compared with the traditional medical metals, Mg alloy has series of better properties: Mg alloy has a smaller density [21], which is the lightest of all metal alloys up to now; the density of Mg and its alloy is very close to that of human bone [22]; Mg and its alloy is the alloy which is found to be the closest to the elastic modulus of human bone, which can effectively alleviate the “stress shielding” effect caused by the large difference between the elastic modulus of implant materials and the elastic modulus of human bone, further avoiding inadequate growth of the new bone tissue without stimulation due to the great difference of mechanical properties [23]; Mg is an essential element of the human body [24], which can be degraded and absorbed with the self-healing of the human body. Therefore, Mg alloy implant treatment does not need a second operation, which can largely exempt patients from the pain of surgery again. Among the Mg alloys, Mg lithium (Li) alloy may be important for patients with osteosarcoma after the operation. Series of studies have demonstrated that the ability of Mg and Li to promote bone healing [25–28]. Wang et al. culture human osteosarcoma MG-63 cells with the degradation products of Mg alloy to observe the corrosion products produced in the degradation process of Mg alloy and the influence of the change of pH value on human osteosarcoma, so as to study the effect of Mg alloy on bone tumor [29]. Li element was also found to have a significant inhibitory effect on osteosarcoma [30, 31]. Thus, in this study, we prepared the Mg–*x*Li–Zinc (Zn) series alloys (*x* = 3 wt%, 6 wt%, 9 wt%) to investigate the influence of Li gradient change on the structure and properties of the alloy, and their functions of inhibiting osteosarcoma and promoting bone growth, for potential application of orthopedic implant materials.

## Materials and methods

### Fabrication and characterization of the Mg–*x*Li–Zn alloys

The Mg–*x*Li–*y*Zn alloys (*x* = 3 wt%, 6 wt%, 9 wt%; *y* = 1 wt%) produced by Shangjie Chinalco Research Institute were cut into small cube samples with their size of 10 mm × 10 mm × 5 mm, and then subtly polished with metallographic abrasive paper of 100#, 200#, 400#, 600#, and 1000#, successively [32], followed with the polishing machine until the sample is bright and free of scratches [33]. The microstructure of polished samples was observed by metallurgical microscopy (DM4000M, Leica, Germany) after corrosion with 4 vol% nitric acid and alcohol for a proper time, and a random visual field from the samples were taken to analyze the of Mg–*x*Li–Zn alloys with different Li content on the microstructure. The phase composition of the Mg–*x*Li–Zn alloys was identified by X-ray diffraction (XRD) [34]. The fracture morphology and corrosion morphology of Mg–*x*Li–Zn alloys were observed by scanning electron microscopy (SEM, Quanta 200, FEI, USA), further matching energy dispersion spectrometer (EDS) to analyze the distribution and content of elements in alloy structure [35]. The chemical properties of corrosion products on the Mg–*x*Li–Zn alloys surfaces were tested by X-ray photoelectron spectroscopy (XPS, AXIS Supra, Shimadzu, Japan), and the binding states of Mg2p, Li1s, O1s, and C1s were determined by high-resolution narrow scan method [36]. The tensile strength, yield strength (YS), and elongation of the Mg–*x*Li–Zn alloys at room temperature were measured by a universal testing machine (AG-IC 50KN, SHIMADZU, Japan) with the tensile rate of 1 mm/min [37].

To investigate the degradation behavior of the Mg–*x*Li–Zn alloys, their electrochemical properties in simulated body fluid (SBF) were tested by using the RST5200 electrochemical workstation (Rhiruisi, Zheng Zhou) according to the previous method [38]; The pH values of the α-MEM medium immersed by the alloys (1.25 cm^2^/ml) after 3 h, 5 h, 24 h, 72 h, 120 h, and 168 h were determined [39], and the Mg^2+^, Li^+^, Zn^2+^ ions in the α-MEM medium were also quantified [35]; Corrosion morphology of the alloys immersed in the α-MEM medium for 24 h, 72 h, 120 h, and 168 h were observed by SEM, and the corrosion products were analyzed by EDS and XPS.

### Cell tests of the Mg–*x*Li–Zn alloys

#### MG-63 cell culture

In order to investigate the inhibition of Mg–*x*Li–Zn alloys on osteosarcoma, the MG-63 cell line (a kind of osteosarcoma) were cultured with the extract solution (α-MEM medium immersed by the alloys for 24 h, 72 h, 120 h, and 168 h) of the Mg–*x*Li–Zn alloys with an initial cell density of 5 × 10^4^ cells/ml for 1 day, 3 days, 5 days, and 7 days, respectively. The viability of MG-63 cultured with each extract solution was examined and calculated by a typical MTT assay [40]. The alkaline phosphatase (ALP) activity of MG-63 cultured with each extract solution was also examined to evaluate the cell behavior [41].

#### MC3T3 cell culture

Osteoblasts play an important role in the process of bone healing [42]. Therefore, MC3T3 cell line (Osteoblasts) were cultured with the extract solution (α-MEM medium immersed by the alloys for 24 h, 72 h, 120 h, and 168 h) of the Mg–*x*Li–Zn alloys with an initial cell density of 5 × 10^4^ cells/ml for 1 day, 3 days, 5 days, and 7 days, respectively. The viability of MC3T3 cultured with each extract solution also was investigated by the MTT method.

### Statistical analysis

All of the values are presented as mean ± standard deviation using Origin8 software. Statistical analyses were performed by *t*-test.

## Results and discussion

### Characterization of the Mg–*x*Li–Zn alloys

**Fig. 1:**
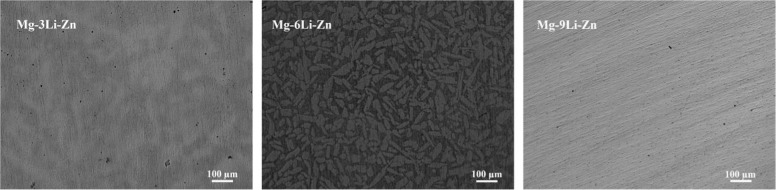
Metallurgical microscopy images of Mg–3Li–Zn, Mg–6Li–Zn, and Mg–9Li–Zn alloys

Figure 1 showed the metallographic microstructure of Mg–*x*Li–Zn alloy: it could be seen from the figures that Mg–3Li–Zn alloy (*x* = 3 wt%) was composed of a single coarse dendrite structure without precipitates; the Mg–6Li–Zn alloy (*x* = 6 wt%) was composed of matrix and strip phase, and the strip phase was uniformly distributed on the matrix and had a relatively large volume; the Mg–9Li–Zn alloy (*x* = 9 wt%) had a single fine dendrite structure.

**Fig. 2:**
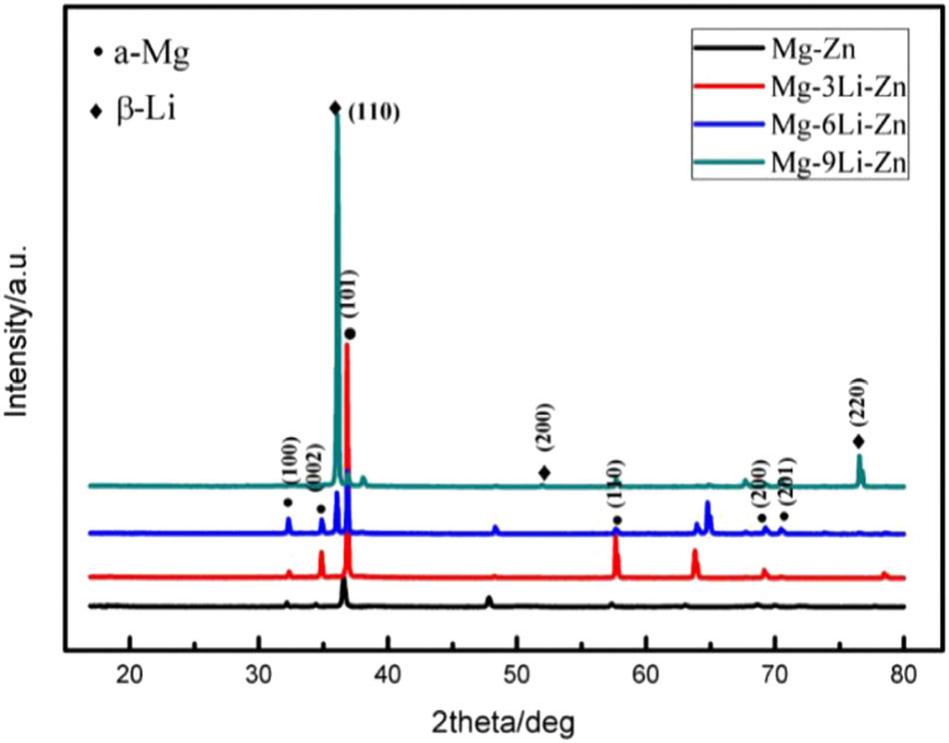
XRD results of Mg–3Li–Zn, Mg–6Li–Zn, Mg–9Li–Zn alloys, and Mg–Zn control

Figure 2 showed the XRD results of as-cast Mg–*x*Li–Zn alloys, and Mg–Zn alloy was selected as the control to analyze the effect of Li content on the phase composition. The phase composition of Mg–*x*Li–Zn alloys was different due to different Li content: when the Li content of the alloy was 3 wt%, the diffraction peak position of the alloy was exactly the same as that of Mg–Zn alloy, which was a single α-Mg phase; When the Li content of Mg–*x*Li–Zn alloy was 6 wt%, the diffraction peak of β-Li phase appeared obviously at the diffraction angle of 36°, and here the phase group of Mg–6Li–Zn alloy became the dual-phase structure of α-Mg and β-Li; when the alloy was Mg–9Li–Zn, only β-Li phase diffraction peaks with diffraction angles of 36°, 52.4°, and 77° appeared in the pattern, so when the Li content was 9 wt%, Mg–*x*Li–Zn alloy was a single β-Li phase. Thus, the results reflected in the XRD pattern are consistent with the results of the metallographic micrograph in Fig. 1.

**Fig. 3:**
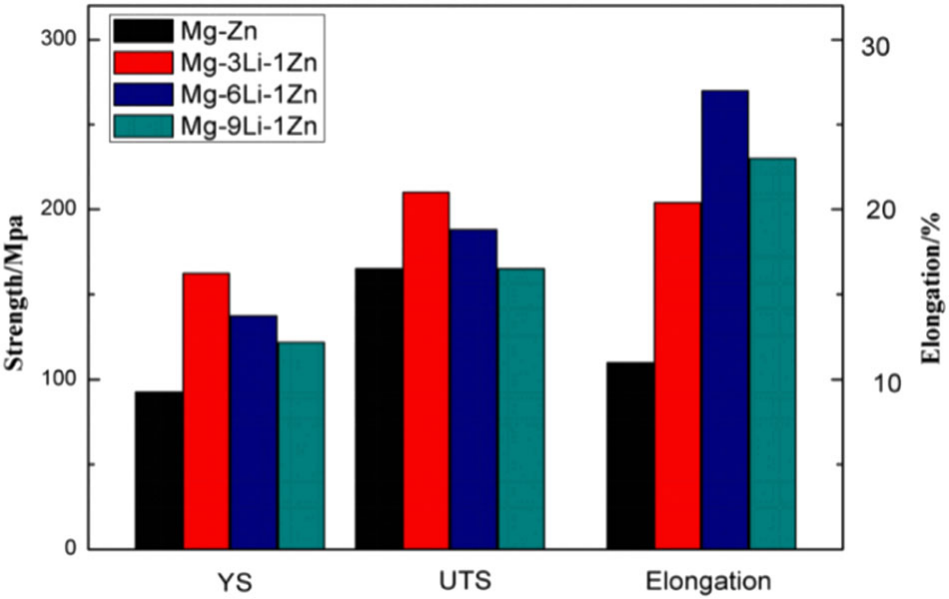
Tensile properties of as-cast Mg–*x*Li–Zn alloy

Human natural bone has certain strength and toughness. If the mechanical properties of the implant material are too different from those of natural bone, it will affect the normal growth of new bone tissue. Therefore, the mechanical properties of the alloy are also an important factor in the selection of implant materials [43]. In this study, the tensile properties of Mg–*x*Li–Zn alloy were measured to study the effect of Li content on tensile properties of the alloy, so as to determine whether Mg–*x*Li–Zn alloy with Li content has mechanical potential as bone implant material. In Fig. 3, the YS, tensile strength (UTS), and elongation of as-cast Mg–*x*Li–Zn alloy are higher than that of Mg–Zn. With the increase of Li content, the YS and tensile strength of Mg–*x*Li–Zn alloy decreased: when Li content was 3 wt%, the elongation of an alloy is the lowest; when Li content was 6 wt%, the elongation of the alloy is the highest. This is because, with the increase of Li content, Mg–*x*Li–Zn alloy has changed from a single α-Mg phase (Mg–3Li–Zn) with close-packed hexagons to a mixed α-Mg/β-Li structure (Mg–6Li–Zn) with close-packed hexagons and BCC, and then to a single β-Li phase (Mg–9Li–Zn) with BCC. The tensile strength and elongation of the alloy change with the change of grain and phase in the alloy. The hardness of the BCC β phase is smaller than that of the HCC α phase, which plays a softening role and is easier to be activated and sliding at room temperature. Therefore, the elongation of the alloy increases with the increase of Li content, and the elongation of the Mg–6Li–Zn alloy was the best because of its dual-phase structure. The change of strength is just the opposite: With the increase of Li content, the strength of alloy decreases.

**Fig. 4:**
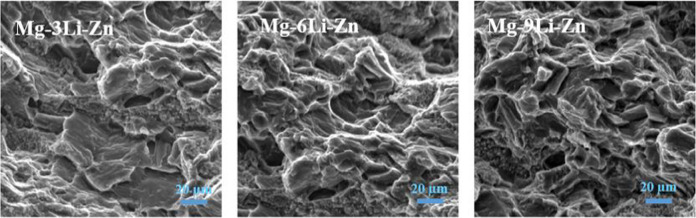
Tensile fracture morphology of Mg–3Li–Zn, Mg–6Li–Zn, and Mg–9Li–Zn alloys

Figure 4 showed that there were dimples and tearing edges in the tensile fracture of Mg–*x*Li–Zn alloy. It was proved that the Mg–*x*Li–Zn alloy produced plastic deformation during the tensile process, indicating that the fracture mode of as-cast Mg–*x*Li–Zn alloy was a mixed fracture with ductile fracture as the main way. In addition, with the change of Li content, the tearing edge in the tensile fracture also had a slight change: the dimple of Mg–3Li–Zn fracture was slightly less than that of the other two alloys, and a slight necking could be seen on the macro fracture of Mg–6Li–Zn; such a metallographic structure showed that the toughness of Mg–6Li–Zn alloy was better than that of Mg–9Li–Zn alloy, and further superior to that of Mg–3Li–Zn alloy (i.e., Mg–6Li–Zn alloy was best, followed by Mg–9Li–Zn alloy, Mg–3Li–Zn alloy the worst.).

**Fig. 5:**
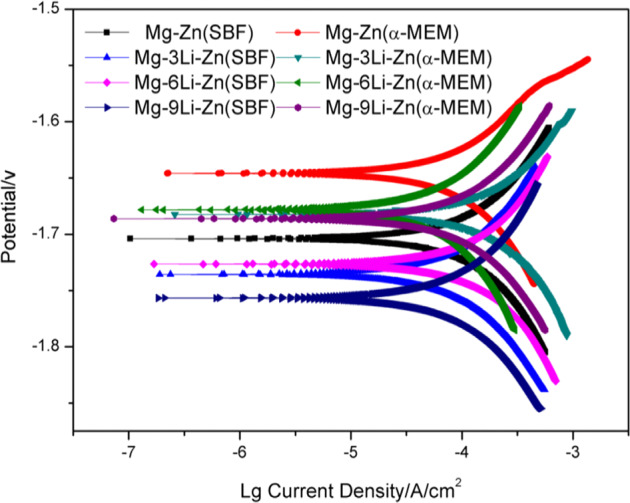
Polarization curves in SBF and α-MEM of Mg–3Li–Zn, Mg–6Li–Zn, Mg–9Li–Zn, and Mg–Zn alloys

In this study, SBF and α-MEM complete media were selected as immersion solutions to determine the difference in corrosion resistance. In Fig. 5, compared with the Mg–Zn alloy, the corrosion potential of the alloy moved along the negative direction as a result of adding Li, indicating that the adding Li had an effect on the corrosion resistance of the alloy; in the as-cast Mg–*x*Li–Zn alloy, with the increase of Li element, the corrosion potential of the alloy first moved to a positive direction and then moved to the negative direction in a large-scale. According to the theory, the difficulty of corrosion of alloy can be reflected by the corrosion potential of alloy: the higher the corrosion potential is, the more difficult it is to be corroded; the corrosion rate of the alloy can be judged by observing the corrosion current density of the alloy: the higher the corrosion current density is, the faster the alloy corrosion is. Figure 5 and Table 1 showed that the corrosion potential (*E*_*corr*_) of Mg–*x*Li–Zn alloy was lower than that of Mg–Zn alloy, and the corrosion current density (*I*_*corr*_) was greater than that of Mg–Zn alloy, indicating the overall corrosion resistance of Mg–*x*Li–Zn alloy was worse than Mg–Zn. In addition, the corrosion potential of Mg–*x*Li–Zn alloy in the α-MEM medium was higher than that of alloy in SBF, which indicated that the alloy slowed down the corrosion process in α-MEM. The reason may be that the medium contains serum, amino acid, protein, and other components, so the degradation product layer formed by the interaction between these complex components and the alloy inhibited the degradation process of Mg–*x*Li–Zn alloys. When the immersion medium is SBF, Mg–6Li–Zn possessed a higher corrosion potential and a lower current density compared to the other samples, suggesting the best corrosion resistance in the Mg–*x*Li–Zn alloys. The polarization curves of the three alloys gradually coincided in the anodic polarization region and showed differences in the cathodic polarization region, which indicated that the corrosion form of Mg–*x*Li–Zn alloys was mainly hydrogen evolution.

**Table 1:** Corrosion potential (*E*_*corr*_) and current density (*I*_*corr*_) of Mg–3Li–Zn, Mg–6Li–Zn, Mg–9Li–Zn, and Mg–Zn alloys

Alloys	SBF		α-MEM	
	*E*_*corr*_ (V)	*I*_*corr*_ (A/cm^2^)	*E*_*corr*_ (V)	*I*_*corr*_ (A/cm^2^)
Mg–Zn	−1.7039	9.631 × 10^−5^	−1.6459	4.504 × 10^−5^
Mg–3Li–Zn	−1.7357	1.252 × 10^−4^	−1.6826	1.985 × 10^−4^
Mg–6Li–Zn	−1.7266	1.116 × 10^−4^	−1.6784	6.695 × 10^−5^
Mg–9Li–Zn	−1.7567	1.408 × 10^−4^	−1.6862	1.153 × 10^−4^

**Fig. 6:**
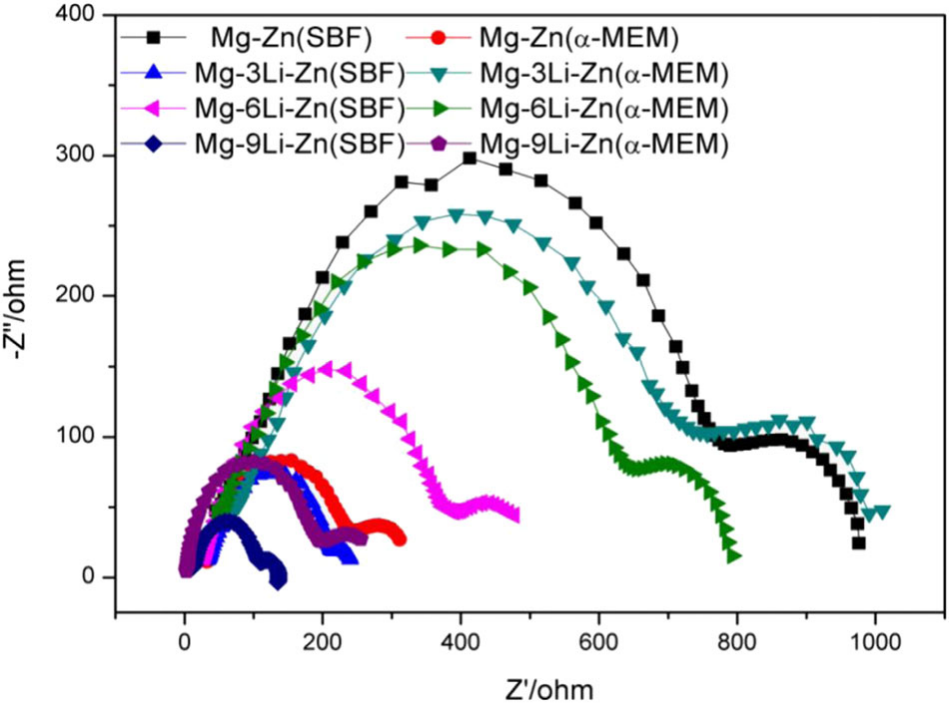
EIS spectra of Mg–3Li–Zn, Mg–6Li–Zn, Mg–9Li–Zn, and Mg–Zn alloys

The radius of arc resistance in EIS spectra can reflect the corrosion rate of alloy: the larger the radius of arc resistance, the better the corrosion resistance of the alloy [44]. Figure 6 showed that in the SBF the capacitance resistance of Mg–Zn alloy was the largest, suggesting the best corrosion resistance, while with the increase of Li content, the radius of capacitive arc increased first and then decreased, which indicated that the corrosion resistance of the alloy increased first and then decreased; while in the α-MEM, the capacitance resistance showed a trend of: Mg–3Li–Zn > Mg–6Li–Zn > Mg–9Li–Zn and Mg–Zn. The capacitance resistance results indicated that Mg–3Li–Zn and Mg–6Li–Zn might have better corrosion resistance than other alloys.

**Fig. 7:**
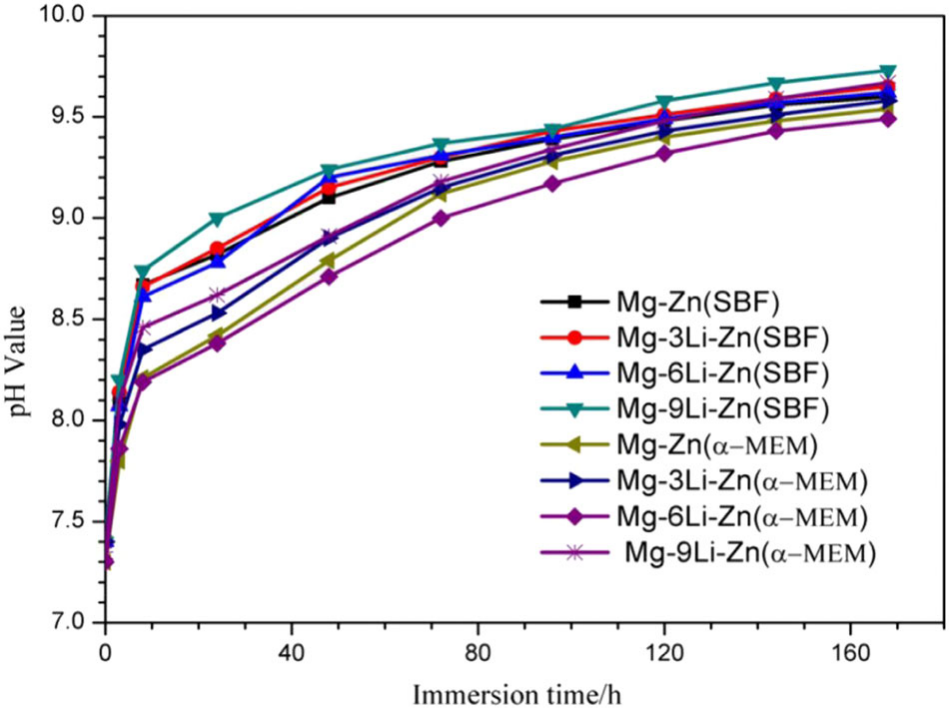
The change of pH value of as-cast Mg–Zn and Mg–*x*Li–Zn alloys during immersion in SBF and α-MEM

The alloy will degrade continuously in the soaking process, and the release of the element ions in the alloy will combine with the element ions in the soaking solution, which will change the pH value of the original soaking solution. The change rate of pH value can also reflect the corrosion rate of alloy to a certain extent, and pH value has a certain influence on cell growth. Figure 7 showed the change of pH in Mg–*x*Li–Zn alloy and Mg–Zn alloy immersed in SBF and α-MEM respectively. With the increase of soaking time, the pH value of a soaking solution is increasing: the pH values of SBF and α-MEM increased linearly in 3 h, followed by a decrease in the rate of pH rise; this means that the corrosion rate of alloy in 3 h is very fast and the rate of corrosion decreases gradually [45]. In the beginning, the pH value of SBF increased faster than that of pH in α-MEM medium, and the pH value of SBF began to stabilize before and after 72 h, while the pH value of α-MEM medium increased slowly in the early stage, and accelerated to a certain extent after 24 h, and began to stabilize until about 96 h, indicating that different immersion media had different effects on the degradation rate of alloy. Moreover, the increasing rate of pH value of the same soaking solution for different alloys was also different, indicating that the Li element also affects the pH value of the soaking solution.

**Fig. 8:**
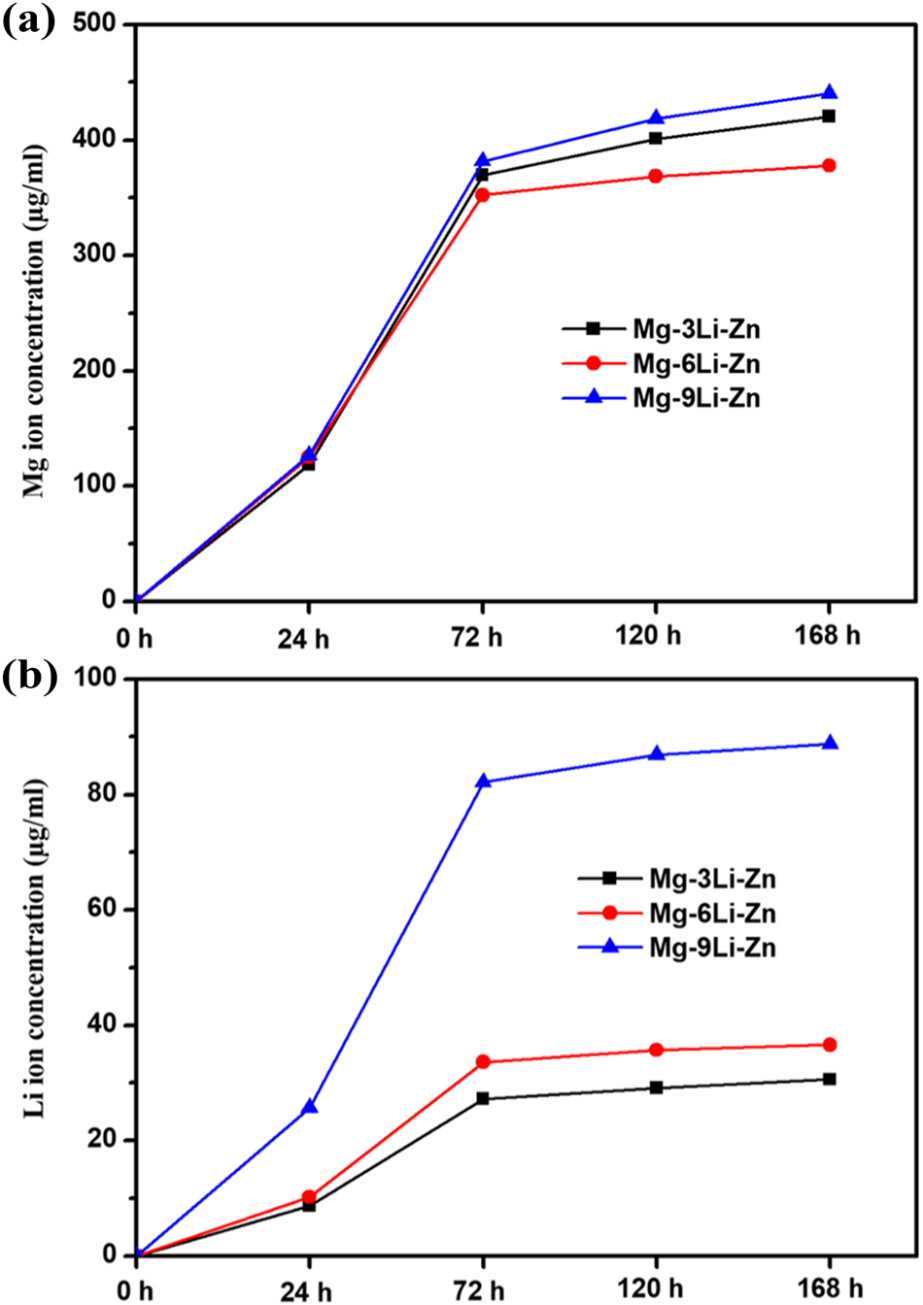
The changes of (**a**) Mg^2+^ and (**b**) Li^+^ ions concentration of as-cast Mg–*x*Li–Zn alloys during immersion in α-MEM

During immersion, corrosion of Mg–*x*Li–Zn alloy is accompanied by the release of ions. With the prolongation of the degradation time, the Mg and Li elements in the α-MEM medium also accumulate and increase, which has a crucial effect on cell survival, proliferation, and apoptosis. Figure 8 showed that the concentrations of Mg^2+^ and Li^+^ in the α-MEM medium increased with the prolongation of soaking time, wherein the concentration of Li^+^ in Mg–9Li–Zn alloy was always at the highest position, and the rate of ion concentration growth was also at the highest level. When the soaking time of the alloy was extended from 24 h to 72 h, the Li^+^ concentration in the Mg–9Li–Zn alloy increased from 25.7 µg/ml to 82.1 µg/ml, an increase of nearly four times, while Li^+^ concentration in Mg–3Li–Zn alloy and Mg–6Li–Zn alloy increased from 8.7 μg/ml and 10.2 μg/ml to 27.2 μg/ml and 33.6 μg/ml, respectively, which verified that the corrosion of Mg–9Li–Zn alloy was quicker than that of Mg–3Li–Zn alloy and Mg–6Li–Zn alloy. The concentration differences of Mg^2+^ and Li^+^ also play different roles in osteoblasts and osteosarcoma cells.

**Fig. 9:**
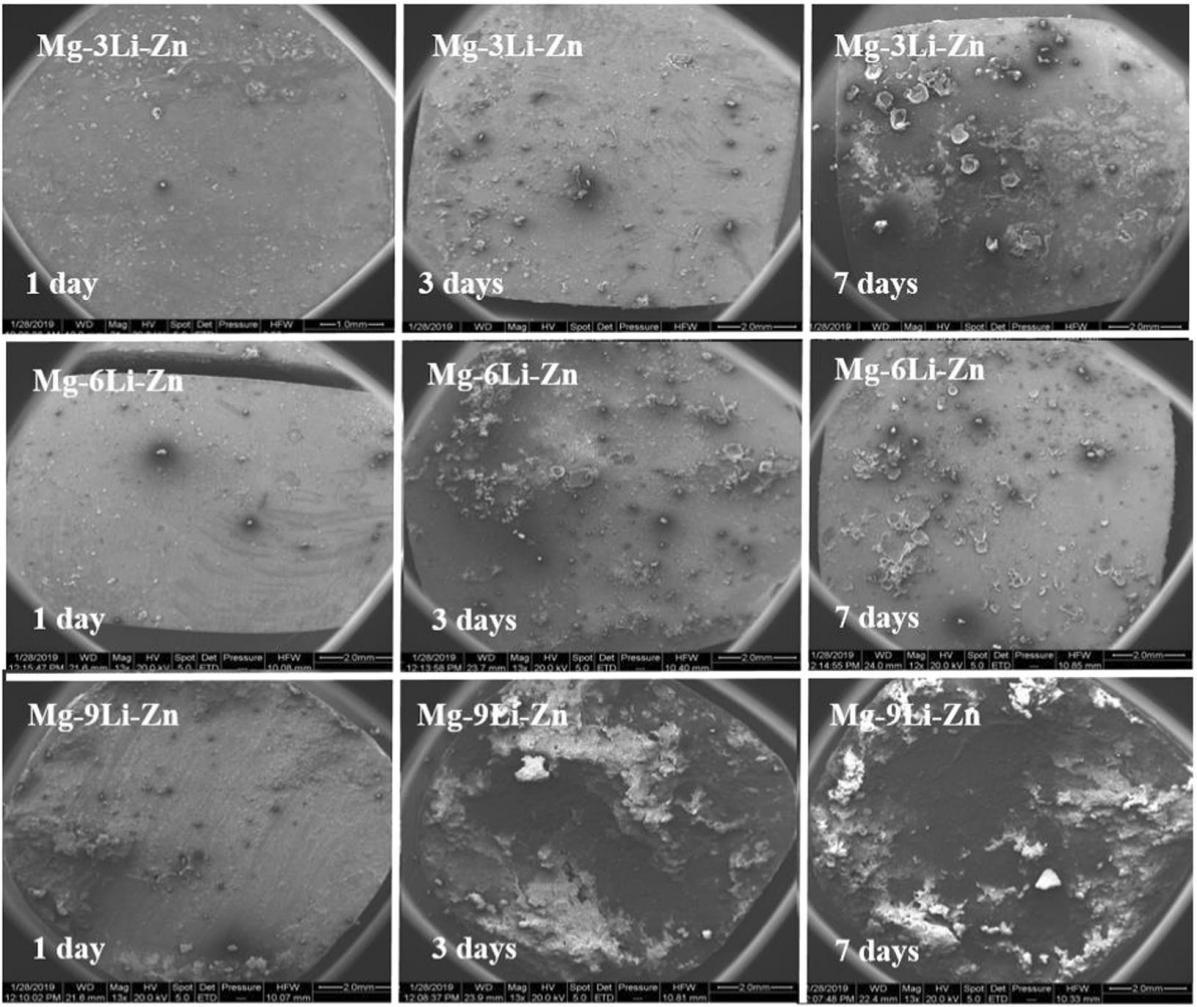
SEM images of as-cast Mg–*x*Li–Zn alloys during immersed in α-MEM for 1 day, 3 days, and 7 days

During the soaking process, the microstructure, grain size, and the second phase of the alloy are different because of the different content of Li in the Mg–*x*Li–Zn alloy. This also leads to the different corrosion rates of the alloy in the medium, so that the corrosion morphology and product will also be different. Figure 9 showed that with the increase of immersion time, the corrosion morphology of alloy surface changed, corrosion products began to accumulate, corrosion layer fell off, and obvious corrosion pit appeared, so the corrosion mode is pitting. HCO_3_^−^/CO_3_^2−^ is contained in α-MEM medium, which can react with Mg–*x*Li–Zn alloy to form corrosion products. When the Li content is small, the corrosion products are mainly Mg(OH)_2_ and MgCO_3_; with the increase of Li content, Li_2_CO_3_ corrosion products are gradually produced. On Mg–9Li–Zn alloy surface, there were many small corrosion pits connecting and converging on the third day and seventh day, which became obvious corrosion pit; the corrosion products were lamellar and falling off continuously, which showed that the corrosion layer of Mg–9Li–Zn alloy was loose and not compact during the corrosion process, which could not slow down the corrosion; with the extension of time, the corrosion pit grew up, which made the contact area between the alloy and the corrosion environment increase, leading to the accelerated degradation of the alloy. On the Mg–3Li–Zn alloy surface, the corrosion of the alloy was not serious and the covered corrosion layer appeared on the first day; on the seventh day, the corrosion pit appeared and the corrosion was not uniform, and the corrosion layer in a small range was lifted and dropped. In contrast, the corrosion products covered the Mg–6Li–Zn surface evenly, and there were small pits and shallow pits at 7 days, and there was no large area of degradation products peeling off, indicating this structure can delay the corrosion speed to a certain extent. All these results indicated that with the increase of Li element content, though Li_2_CO_3_ film gradually appeared in the corrosion products of the alloy, other compounds in the alloy will weaken its protection so that it could not play an effective role in slowing down the degradation.

**Fig. 10:**
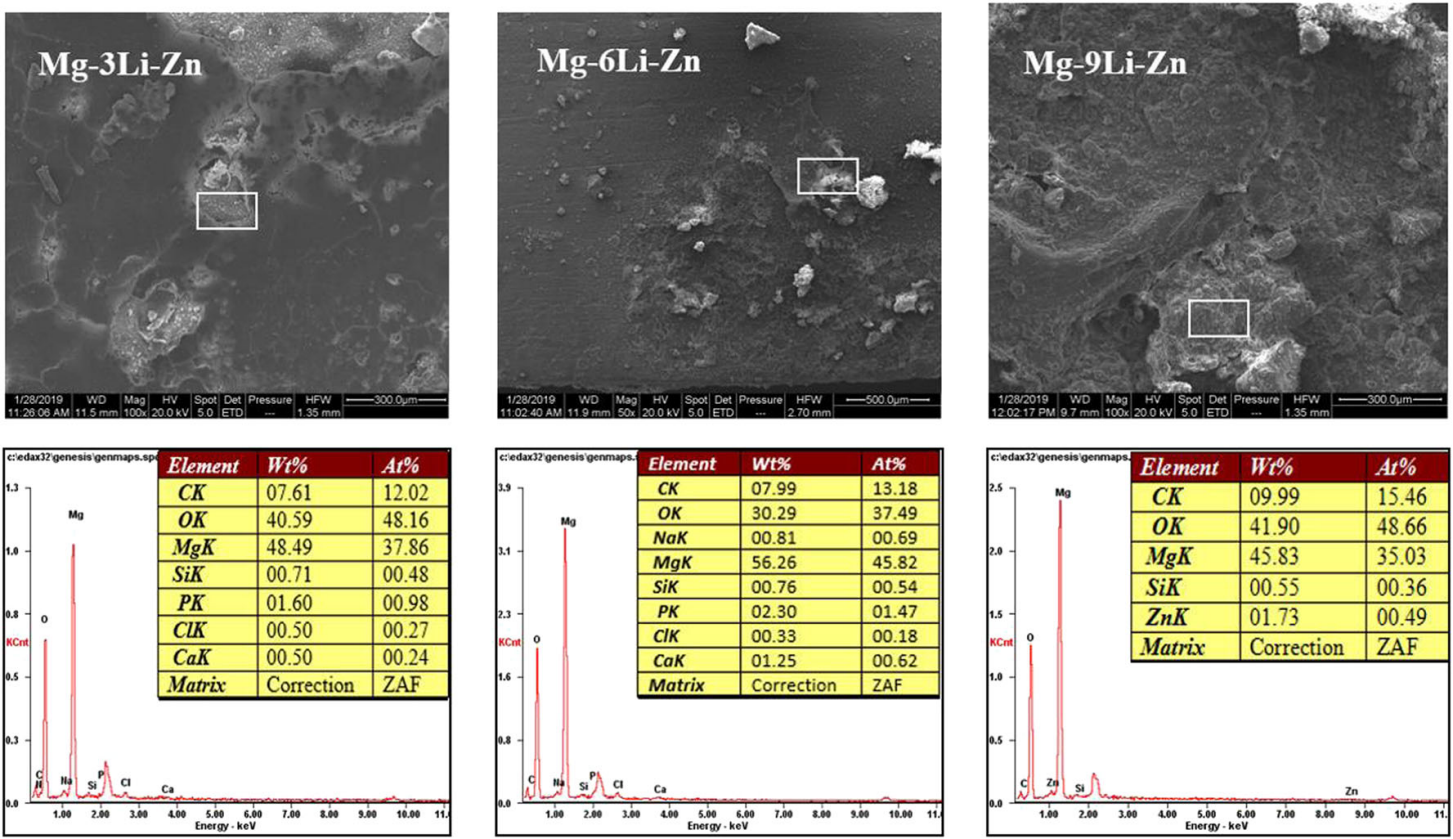
The EDS of the typical area on the as-cast Mg–*x*Li–Zn alloys surfaces after immersed in α-MEM for 7 days

The EDS results in Fig. 10 showed that the content of Mg in the corrosion products was high, forming a corrosion layer mainly composed of Mg, O, and C, which was not conducive to the corrosion resistance of the alloy; wherein, there were Ca and P in the corrosion products of Mg–3Li–Zn and Mg–6Li–Zn alloys, indicating that there may be a Ca/P salt corrosion product layer in the corrosion products of the alloys [46], while the corrosion products of Mg–9Li–Zn alloy did not contain P element, which could not form a dense Ca/P salt corrosion layer, so the corrosion resistance of Mg–9Li–Zn alloy was not as good as that of the former two alloys. According to the change of C content in the EDS, the corrosion products of Mg–3Li–Zn alloy were mainly Mg(OH)_2_, and there was an MgCO_3_ precipitation layer in the corrosion products of Mg–6Li–Zn alloy, which could delay the further degradation of the alloy; with the increase of Li content, the C content in the corrosion products increased from 12.2% to 15.46%, which proved that the Li_2_CO_3_ content in the corrosion products of the alloy also increased, and the Li_2_CO_3_ coating will lose the protective effect on the degradation of the alloy due to the action of other compounds in the alloy [47].

**Fig. 11:**
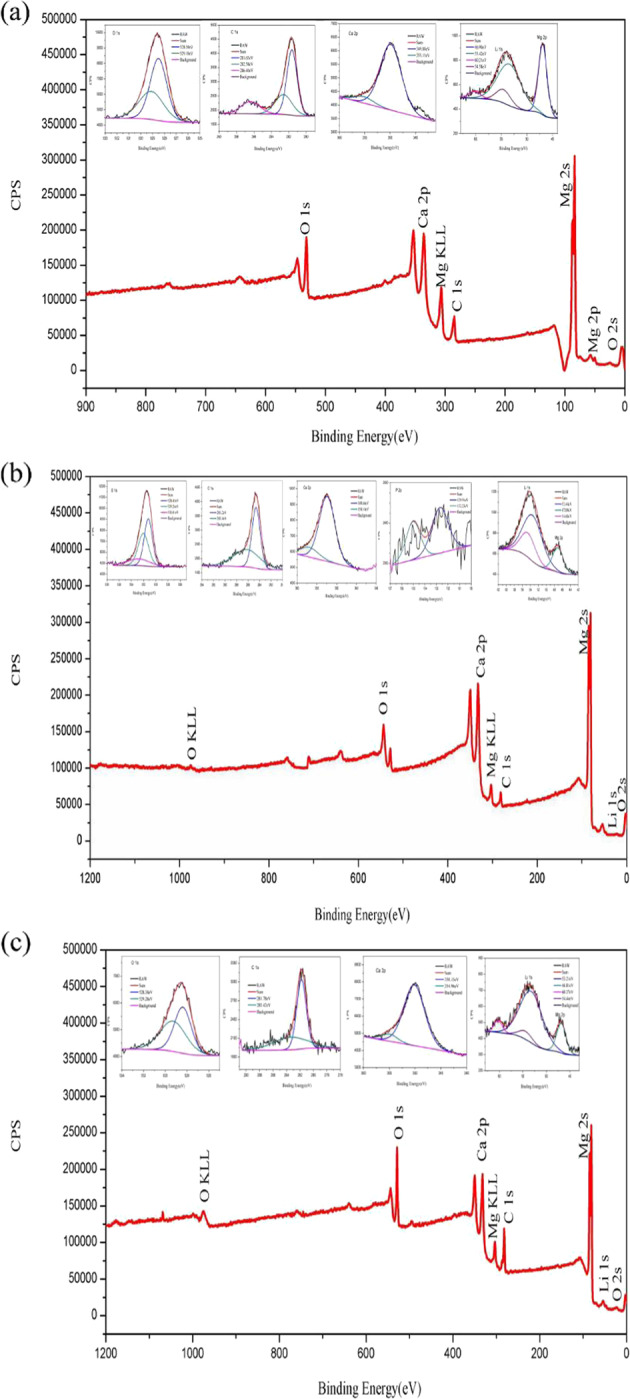
XPS spectrum of (**a**) Mg–3Li–Zn, (**b**) Mg–6Li–Zn and (**c**) Mg–9Li–Zn

The XPS full spectrum of corrosion products of as-cast Mg–xLi–Zn alloys showed that the corrosion products contained Mg, Li, O, C, Ca, and P elements (Fig. 11). By comparison, the corrosion products of Mg–3Li–Zn alloy contain more Mg^2+^, while the Li 1 s peak of corrosion products of Mg–6Li–Zn and Mg–9Li–Zn alloys is more significant. Through further analysis of the detected elements by high-resolution narrow scan, a Li 1s peak assigned to 54.58 eV was detected in the corrosion products of Mg–3Li–Zn alloy, which was accompanied by C 1 s of 286.40 eV and O 1 s of 529.1 eV, indicating that Li_2_CO_3_ was contained in the corrosion products, and the wide peak range of O 1 s indicated that O in the corrosion products had different chemical states, and the main Mg-based compounds were Mg(OH)_2_ or MgCO_3_. There was more Li in the corrosion products of Mg–6Li–Zn alloy: In addition to Li_2_CO_3_, there was also a 53.44 eV Li 1 s peak, which was Li_2_O; in addition, P 2p peaks of 129.91 eV and 132.23 eV appeared in the corrosion products of Mg–6Li–Zn alloy, which may be related to PO_4_^3−^ or HPO_4_^2−^ [48], indicating that Ca/P salt corrosion layer was formed in the degradation process of Mg–6Li–Zn alloy, and this was one of the reasons why the corrosion resistance of Mg–6Li–Zn alloy was better than that of the other two alloys. Figure 11c showed that the Li 1 s peak of corrosion product of Mg–9Li–Zn alloy was more significant: a large amount of Li_2_CO_3_ appeared in the corrosion products, accompanied by the appearance of Li_2_O and Li(OH)_2_, and the interaction of various compounds in the corrosion products of the Mg–9Li–Zn alloy caused the corrosion layer to lose the protective effect on the alloy. The XPS results showed that the content and chemical value of C, O, Li, and Mg in the corrosion products changed with the change of Li content in the alloy, which further affected the degradation of as-cast Mg–*x*Li–Zn alloys.

### Biological functions of the Mg–*x*Li–Zn alloys

**Fig. 12:**
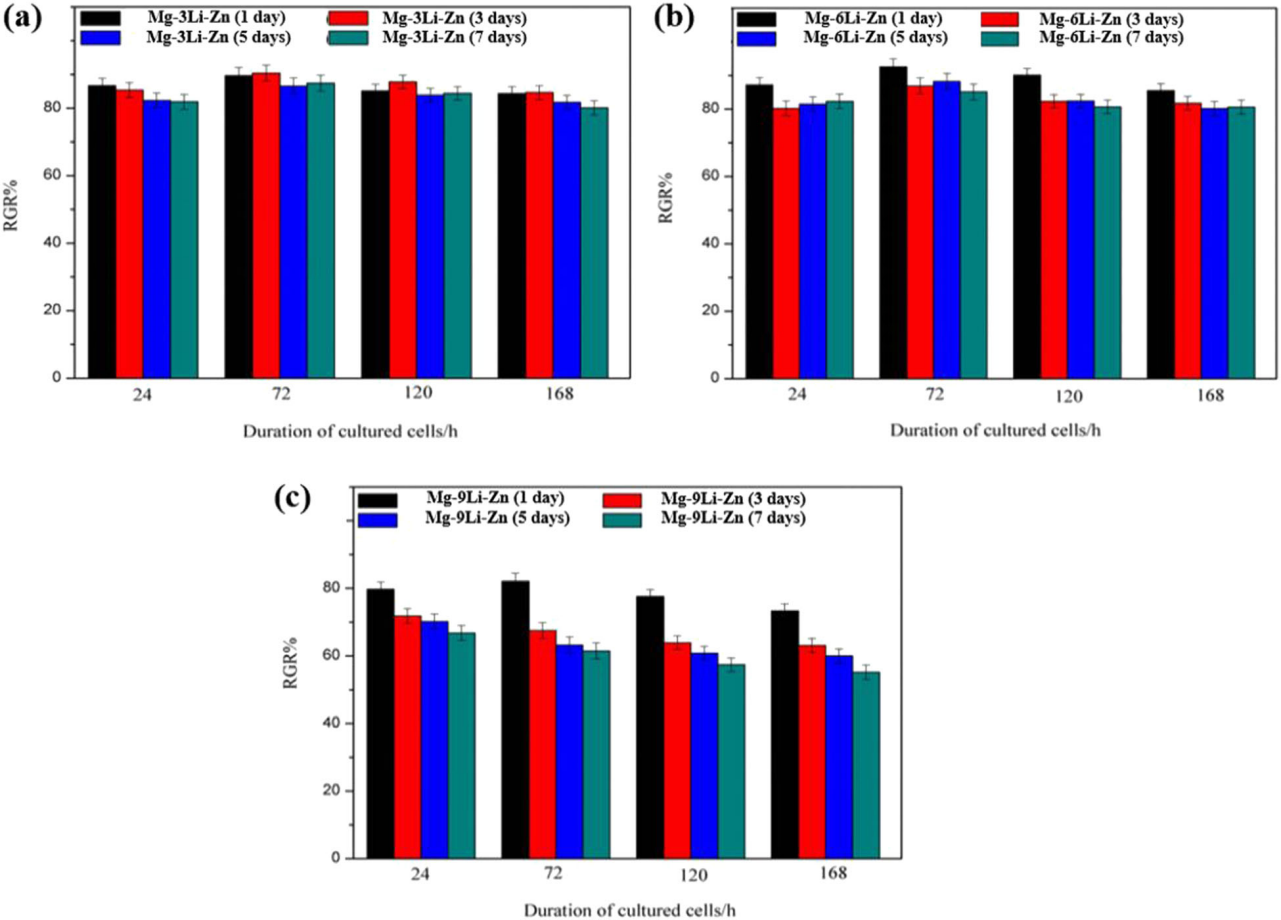
Viability of MG-63 cells of (**a**) Mg–3Li–Zn, (**b**) Mg–6Li–Zn and (**c**) Mg–9Li–Zn (mean ± SD, *n* = 3)

**Fig. 13:**
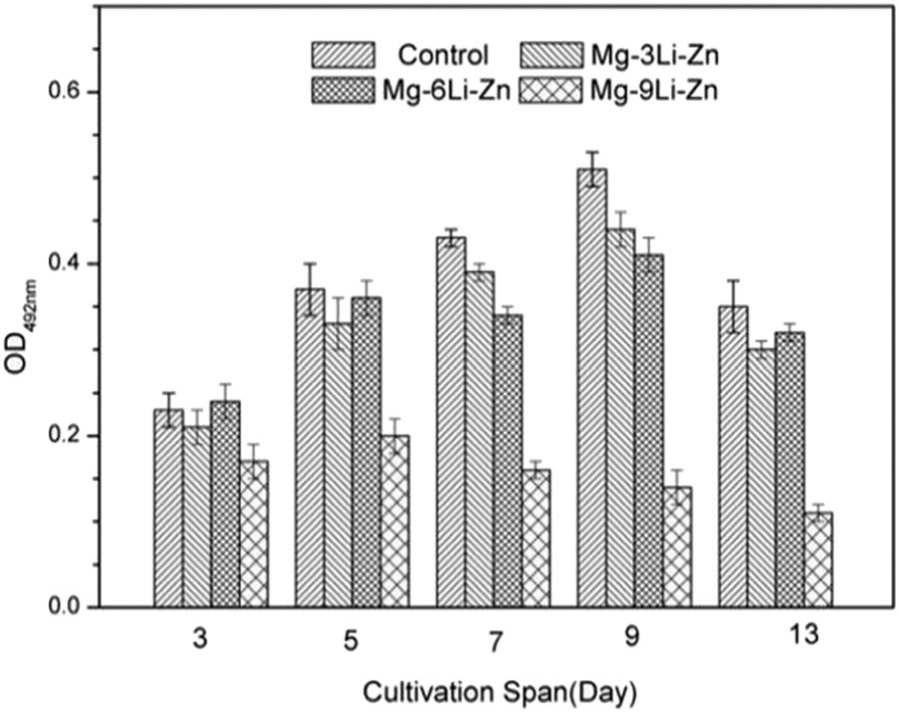
ALP activity of MG-63 in the Mg–3Li–Zn, Mg–6Li–Zn, Mg–9Li–Zn, and control (positive) group (mean ± SD, *n* = 3)

Figure 12 depicted that the Mg–*x*Li–Zn presented a trend of inhibiting osteosarcoma cell (MG-63): Mg–9Li–Zn superior to Mg–6Li–Zn, and superior to Mg–3Li–Zn; as time went on (including degradation time of Mg–*x*Li–Zn alloys and interaction time with MG-63 cells) the inhibition of MG-63 cells by Mg–*x*Li–Zn alloys gradually increased. Wherein, the MG-63 in the Mg–6Li–Zn group reached a stable apoptosis rate at the third day of interaction, while the apoptosis rate of MG-63 in the Mg–9Li–Zn group kept rising till the seventh day, which indicated their good ability on inhibiting osteosarcoma. The ALP activity results of MG-63 cells displayed the consistent trend with the MTT detection: Mg–9Li–Zn superior to Mg–6Li–Zn, and superior to Mg–3Li–Zn; all the Mg–*x*Li–Zn alloys showed strong functions on inhibiting osteosarcoma (Fig. 13).

**Fig. 14:**
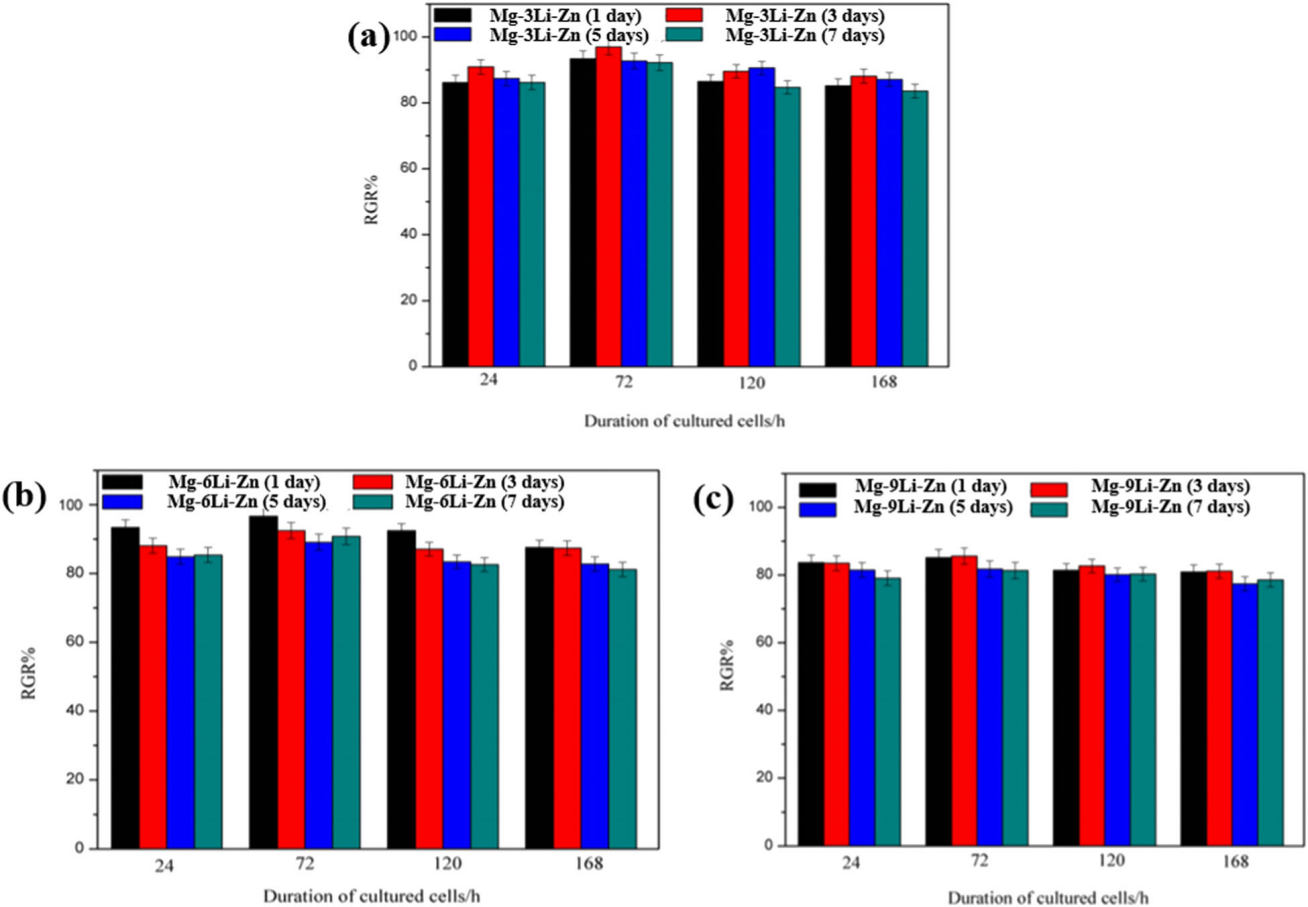
Viability of MC3T3 cells of (**a**) Mg–3Li–Zn, (**b**) Mg–6Li–Zn and (**c**) Mg–9Li–Zn (mean ± SD, *n* = 3)

Osteoblasts belong to alkaline tolerant cells [49, 50]. Therefore, osteoblasts cultured with Mg–*x*Li–Zn alloy extract may not lead to cytotoxicity (viability > 80%). Figure 14 proved our speculation: MC3T3 cells (Osteoblasts) in all Mg–*x*Li–Zn alloy groups showed a viability > 80%, indicating no cytotoxicity caused by the alloys. Wherein, Mg–3Li–Zn, and Mg–6Li–Zn group promoted the proliferation of MC3T3 in a certain concentration of degradation products (influenced by the degradation time of Mg–*x*Li–Zn alloys and interaction time with MC3T3).

## Conclusion

In this contribution, we developed a series of Mg–*x*Li–Zn alloys to investigate their mechanical property, degradability, and influence on osteosarcoma (MG-63) and osteoblasts (MC3T3) for potential application of bone-implant material. Our data indicated the summary with specific results as follow:

(i) The content of Li decided the microstructure, phase composition, mechanical property, degradability, and biological functions of the Mg–*x*Li–Zn alloys.
(ii) Among these Mg–*x*Li–Zn alloys, Mg–6Li–Zn alloy owned better mechanical property.
(iii) Mg–6Li–Zn and Mg–3Li–Zn showed better biodegradability compared with the other alloys.
(iv) For inhibiting osteosarcoma growth, the Mg–*x*Li–Zn alloys showed a trend of: Mg–9Li–Zn > Mg–6Li–Zn > Mg–3Li–Zn; for promoting osteoblast growth, the Mg–*x*Li–Zn alloys presented a trend of: Mg–3Li–Zn > Mg–6Li–Zn > Mg–9Li–Zn.
(v) Thus, the Mg–6Li–Zn may display a feasible and effective approach to addressing the problems of biodegradable bone implants.
